# Screening and prevention of preterm birth: how is it done in clinical practice?

**DOI:** 10.61622/rbgo/2024rbgo32

**Published:** 2024-04-09

**Authors:** Roberta Bulsing dos Santos, Janete Vettorazzi, Marcos Wengrover Rosa, Ellen Machado Arlindo, Edimárlei Gonsales Valério

**Affiliations:** 1 Universidade Federal do Rio Grande do Sul Porto Alegre RS Brazil Universidade Federal do Rio Grande do Sul, Porto Alegre, RS, Brazil.; 2 Hospital Moinhos de Vento Obstetrics and Gynecology Department Porto Alegre RS Brazil Obstetrics and Gynecology Department, Hospital Moinhos de Vento, Porto Alegre, RS, Brazil.; 3 Hospital de Clínicas de Porto Alegre Gynecology and Obstetrics Department Porto Alegre RS Brazil Gynecology and Obstetrics Department, Hospital de Clínicas de Porto Alegre, Porto Alegre, RS, Brazil.

**Keywords:** Cervical length measurement, Infant, premature, Preterm birth, Screening, Prevention, Gynecologists, Obstetricians, Health knowledge, attitudes, practice, Surveys and questionnaires

## Abstract

**Objective:**

To ascertain how screening for preterm birth is performed among obstetricians working in public and private practice in a middle-income country.

**Methods:**

Cross-sectional study of 265 obstetrician-gynecologists employed at public and private facilities. An online questionnaire was administered, with items designed to collect data on prematurity screening and prevention practices.

**Results:**

The mean age of respondents was 44.5 years; 78.5% were female, and 97.7% had completed a medical residency program. Universal screening (i.e., by ultrasound measurement of cervical length) was carried out by only 11.3% of respondents in public practice; 43% request transvaginal ultrasound if the manual exam is abnormal, and 74.6% request it in pregnant women with risk factors for preterm birth. Conversely, 60.7% of respondents in private practice performed universal screening. This difference in screening practices between public and private practice was highly significant (p < 0.001). Nearly all respondents (90.6%) reported prescribing vaginal progesterone for short cervix.

**Conclusion:**

In the setting of this study, universal ultrasound screening to prevent preterm birth was used by just over half of doctors in private practice. In public facilities, screening was even less common. Use of vaginal progesterone in cervical shortening was highly prevalent. There is an unmet need for formal protocols for screening and prevention of preterm birth in middle-income settings.

## Introduction

Prematurity remains a major public health problem worldwide. In 2012, the World Health Organization published the document "Born too soon", containing alarming data on preterm birth: about 15 million children are born preterm annually, of which 1.1 million die as a result of the consequences of preterm birth.^(1)^

Brazil is the ranks tenth in the world by highest absolute number of preterm births.^(1)^ It is evident that important aspects of prenatal care are failing in Brazil, such as assessment of maternal-fetal risk by clinical history, physical examination, and transvaginal ultrasound with cervical measurement. The role of the prenatal care team is to assess and offer all of the tools available to prevent this outcome. A thorough history and physical examination can help predict 38% of preterm births. Combined with transvaginal ultrasound to measure cervical length, this predictive ability rises to 69%.^(2)^

Transvaginal ultrasound, when performed in the second trimester between 18-24 weeks of gestation, has been shown to have a strong positive predictive value for preterm birth risk (75% when cervical length is equal to or less than 25 mm).^(3)^ The use of progesterone in patients with cervical shortening has also been shown cost-effective81in theoretical models, with a number needed to treat of 10-19 to prevent 1 case of preterm delivery or prematurity-related outcome.^(4)^

Considering the low cost of transvaginal ultrasound compared to the costs of a preterm birth, the present study aimed to identify how screening and prevention of preterm birth are performed in real-world clinical practice in a middle-income setting.

## Methods

Cross-sectional study of gynecologists and obstetricians (OB-GYNs) in public and private practice in the state of Rio Grande do Sul, Brazil (population 10.69 million people),^(5)^ where, according to data from the State Health Department, 117,100 deaths and 124,400 births occurred in 2021.^(6)^ An anonymous online questionnaire (Quick Tap Survey) was designed to collect data on how respondents screen for preterm birth and how they manage leading risk factors for preterm birth. Questionnaires were sent via the institutional e-mail list of the Rio Grande do Sul Association of Obstetrics and Gynecology, the state's official specialty board. The items comprised data on general profile, training, practice setting, academic affiliations, screening practices, and management of risk factors for preterm birth. Screening for preterm birth was defined as *clinical* when it comprised obstetric history and physical examination alone, and *universal* when it also included evaluation of cervical length by transvaginal ultrasound for all patients.

Questionnaires completed by practicing physicians who had cared for pregnant women in the year 2019 were included. Considering the universe of potential respondents (2,405 practicing obstetricians registered with the Rio Grande do Sul Regional Board of Medicine), a screening prevalence of 81% as described in the literature, an absolute error rate of 0.05, and 95% confidence limits, the minimum sample size was defined as 216 respondents. With 10% extra to account for attrition, the final sample size was set at 238.^(7)^

Data were compiled in SPSS 20.0. The primary analysis was descriptive, consisting of estimation of prevalence. Quantitative data are expressed as medians and interquartile ranges, as the Kolmogorov–Smirnov test rejected the assumption of normality. Qualitative data are expressed as absolute (n) and relative (%) frequencies. The chi-square or Yates-corrected chi-square method was used to test for association between categorical variables, and the Mann–Whitney *U* test for quantitative variables. The significance level was set at 5%.

This study was conducted in accordance with all applicable guidelines and regulations on human subject research in Brazil as per National Health Council Resolution 510/2016. Prior ethical approval was obtained from the Research Ethics Committee 3.128.006 where the study was conducted (CAAE 02901618.6.0000.5330).

## Results

E-mails with the questionnaire were sent to all 2,042 gynecologists and obstetricians registered in the state. Of these, 265 were completed and included in the study. The mean age of respondents was 44.5 years; 78.5% were female and only 2.3% had not completed an OB-GYN residency program. Most respondents (60.4%) practiced both privately and in the public Unified Health System; 35.8% were involved in some form of academic activity; 50.9% practiced in the state capital, Porto Alegre; and 52.5% take obstetric call on a regular basis (Table 1).

**Table 1:** Characteristics of respondents

	In private practice n(%)	In public practice n(%)	Both n(%)	Professor/instructor n(%)	Practices in state capital n(%)	Practices in greater capital region n(%)	Practices elsewhere in state (medical school in town) n(%)	Practices elsewhere in state (no medical school in town) n(%)
OB-GYNs,	245(92.5)	180(67.9)	160(60.4)	95(35.8)	135(50.9)	55(20.8)	57(21.5)	57(21.5)
Age, mean ± standard deviation	45.1±11.4	42.3±11.3	42.9±11.3	45.5±11.7	45.5±10.8	43.0±12.5	43.3±13.1	42.6±9.8
Female	190(77.6)	139(77.2)	121(75.6)	66(69.5)	107(79.3)	45(81.8)	46(80.7)	43(75.4)
Completed medical residency	240(98.0)	178(98.9)	159(99.4)	94(98.9)	130(96.3)	55(100.0)	55(96.5)	57(100.0)
Years of practice, mean ± standard deviation	20.4±11.5	17.3±11.4	18.0±11.4	20.7±11.8	21.0±10.9	17.7±12.5	17.9±13.1	18.0±10.2
Takes obstetric call	124(50.6)	128(71.1)	113(70.6)	64(67.4)	66(48.9)	33(60.0)	32(56.1)	32(56.1)

OB-GYNs - gynecologists and obstetricians

Most performed clinical screening for preterm birth, whether in public (84.7%) or private (96.3%) practice. Universal screening for preterm birth (i.e., including cervical length measurement by transvaginal ultrasound) was performed by only 11.4% of respondents practicing in the public health system. Less than half (43.3%) order a transvaginal ultrasound even if the manual exam is abnormal, but 74.7% request one if patients have a risk factor for preterm birth. In private practice, 60.8% of respondents reported universal screening for preterm birth; 25.4% order an ultrasound if the manual exam is abnormal, and 33.6% order one only for patients with risk factors. Both the prevalence of universal screening and that of clinical screening alone differed significantly between public and private practice (p<0.001, chi-square test with Yates continuity correction). Of those respondents affiliated with a university clinic or other teaching service, only 60.8% reported universal screening, versus 78% of those with no academic affiliation (p = 0.013). Clinical screening was not associated with academic affiliation (99.0% of those practicing in a university-affiliated setting versus 99.4% of those with no such affiliation; p = 0.999). Among providers who practice in the state capital, 74.2% reported doing universal screening, versus 68.6% of those practicing elsewhere in the state, with no significant association (p = 0.464). The prevalence of clinical screening was exactly the same in respondents who practice in the state capital and in those who practice elsewhere (99.2%). There was no significant association (p = 1.000). There was no difference in years of practice between those who perform universal screening and for those who do not (median 16 versus 22 years, p = 0.075). Likewise, there was no difference in years of practice between those who perform clinical screening and those who do not (median 18 versus 11 years, p = 0.297). Regarding the timing of cervical length measurement in singleton pregnancy, 85.3% of respondents ordered ultrasound in the second trimester and 24.5% ordered it in the first trimester. The most common cutoff point for diagnosis of cervical shortening (72.5% of respondents) was ≤ 25 mm. Of all respondents, only 13.4% of respondents do not order cervical length measurement at all in twin pregnancies (Table 2).

**Table 2:** Cervical length cutoff for diagnosis of short cervix

	Singleton pregnancy n(%)	Twin pregnancy n(%)
≤ 15 mm	9(3.4)	9(3.4)
≤ 20 mm	43(16.3)	48(18.3)
≤ 25 mm	192(72.7)	136(51.7)
≤ 30 mm	15(5.7)	35(13.3)
Does not perform cervical measurement	5(1.9)	31(11.8)
NA/other	1(0.4)	6(1.5)

NA - not applicable

Regarding management of confirmed cervical shortening (Table 3), 37.3% of respondents perform watchful waiting with serial examinations alone in singleton pregnancies, as do 31.6% in twin pregnancies. However, when asked about vaginal progesterone, 90.6% claimed to prescribe it in singleton pregnancies and 85.2% in twin pregnancies. Corticosteroids were prescribed more often for multiple pregnancies (45.5%) than for singleton pregnancies (34.3%). In pregnant women with a previous history of spontaneous preterm delivery, 56.9% of respondents prescribed progesterone regardless of cervix length, all starting on the 14th gestational week or later. Just over half of respondents (53.4%) would not indicate serial monitoring of cervix length in these patients. In patients with a prior history of uterine surgery or malformation, 75.5% of respondents would prescribe progesterone only after diagnosis of cervical shortening, while 18.1% would prescribe it regardless.

**Table 3:** Management of short cervix

	Singleton pregnancy n(%)	Twin pregnancy n (%)
Watchful waiting	100(37.7)	83(31.6)
Recommends time off work	121(45.7)	173(65.5)
Recommends hospital admission	8(3)	26(9.8)
Does nothing	5(1.9)	11(4.2)
Prescribes oral progesterone	13(4.9)	13(4.9)
Prescribes vaginal progesterone	240(90.6)	225(85.2)
Cerclage	44(16.6)	48(18.2)
Pessary	53(20.0)	63(23.9)
Prescribes steroids for pulmonary maturation	91(34.3)	120(45.5)

## Discussion

In the United States, Medicaid Health Plans of America suggests routine cervical length screening for all women between 18 and 24 weeks of gestation,^(7)^ as this has proven to be a cost-effective public health action, reducing outcomes such as neonatal death and preterm birth with long-term neurological deficits.^(7-9)^

Martell et al.^(7)^ administered a questionnaire to physicians who treated pregnant patients in the United States in 2016. Of an estimated sample of 30,000 OB-GYNs with American Medical Association membership, 500 complete questionnaires were obtained and analyzed (sample calculation and statistical power calculation were not performed for this study). Compared to our sample, this study was less representative; we obtained 265 complete questionnaires from a potential population of 2,405 obstetricians versus 500 questionnaires for a population of 30,000 providers. We covered only one state of Brazil, while Martell et al.^(7)^ included 9 demographic regions in the United States. Our population of physicians also had a much higher rate of academic affiliation (35.8% versus 13%). In the Martell et al.^(7)^ sample, universal screening for preterm birth was performed by 81% of respondents. In our study, 60.8% of respondents in private practice ordered transvaginal ultrasound for all patients, versus only 11.4% of those in public practice. In a study published in 2017, Berghella et al.^(10)^ evaluated the effect of knowledge of the cervical length in preventing spontaneous preterm birth in singleton pregnancies presenting with threatened preterm labor. There is a significant association between knowledge of cervical length and lower incidence of spontaneous preterm birth and later gestational age at delivery in symptomatic singleton gestations with threatened preterm labor. Navathe et al. (2019)^(11)^ analyzed birth cohorts of preterm infants (gestational age 23 weeks to 33 weeks 6 days) in the year 2011 the year before implementation of a protocol for universal ultrasound screening of cervical length and in the year 2014. There were a significant decrease in the incidence of threatened preterm labor once the protocol was well established, from 11% in 2011 to 6.7% in 2014.^(11)^

In the Martell et al.^(7)^ survey, 490 physicians (98%) reported treating their patients with a diagnosis of short cervix, and even though the U.S. protocol advises the use of vaginal progesterone, 45% prefer to use synthetic intramuscular progesterone. The vast majority of respondents in our study (90.6%) prescribe progesterone for vaginal administration; only 4.9% prescribe oral forms. No respondent mentioned the use of intramuscular synthetic progesterone. In 2012, Romero et al.^(12)^ conducted a meta-analysis of 5 studies which evaluated the use of vaginal progesterone in patients classified as at risk for preterm birth. Patients were divided into a progesterone group and a placebo group. Among those who received progesterone, reductions were observed in preterm birth (<28, <33, and <35 weeks), neonatal respiratory distress syndrome, neonatal ICU admission, need for mechanical ventilation, and overall neonatal morbidity and mortality.^(12)^ Norman,^(13)^ in a review article published in 2020, analyzed trials such as PROLONG, OPPTIMUM and PROGRESS, all of which included significant numbers of patients receiving vaginal and intramuscular progesterone. The authors concluded that there was no statistically significant improvement in outcomes related to preterm birth in the groups of patients who used progesterone when compared with the outcomes of patients in the placebo groups.^(13,14)^

The most commonly cited cutoff point for short cervix is 25 mm: 59-62% in the survey by Martell et al.^(7)^ and 72.7% in our sample. It is likely that the cutoff point of 25 mm is most often chosen because studies have shown that, at or below this length, the risk of preterm birth can reach up to 25%.^(3)^

Both in the Martell et al.^(7)^ survey and in our sample, cerclage and pessary were only very rarely used, probably due to the lack of scientific evidence of their effectiveness.

Regarding pessaries and cerclage, a meta-analysis by Jarde et al.^(15)^ was published in the British Journal of Obstetrics and Gynaecology in 2017. The authors included 36 studies (more than 9,000 patients), comparing both methods versus progesterone.^(15)^ While reductions in preterm births (<34 and <37 weeks) and in the number of neonatal deaths were observed among patients prescribed progesterone, there was no such reduction the cerclage group, while in the pessary group results were inconsistent.

In twin pregnancies, 53% of American professionals always prescribe progesterone for patients with a short cervix, while 90.1% of obstetricians in our study do the same.^(7)^

In 2016, Pagani et al.^(16)^ published a study in which they analyzed different cutoff points for cervical length in twin pregnancies as predictors of preterm birth. A total of 940 twin pregnancies were examined by transvaginal ultrasound between 18 and 23 weeks of gestation. The authors found that the optimal cutoff point for prediction of preterm birth in these pregnancies would be ≤ 36 mm.

In a meta-analysis of individual patients published in Ultrasound in 2017, Romero et al.^(17)^ evaluated the use of progesterone in twin pregnancies diagnosed with a short cervix. The meta-analysis included 303 participants with a cervical length < 25 mm, divided into placebo and progesterone groups. There were 31% fewer births at < 33 weeks, overall lower rates of births at < 34, < 35, and < 30 weeks, lower rates of neonatal death, and less need for mechanical ventilation in the progesterone group. The EVENTS study found weak evidence of an interaction between cervix length and use of progesterone, suggesting harm for those patients with cervical length ≥ 30 mm and potential benefit for those with < 30 mm.^(17,18)^

Unlike Brazil, the United States has no publicly funded national health system. Therefore, in the aforementioned studies there was no difference between public and private practice another important factor to be considered when comparing results to those of our study. Our respondents’ attitudes varied considerably between public and private practice, especially concerning the use of screening methods. That 60.8% of obstetricians in private practice reported ordering transvaginal ultrasound for all patients, while only 11.4% of those practicing in the public sector do so, may be explained by the fact that the Brazilian Unified Health System does not cover this procedure in pregnancies classified as low risk, disregarding that lower socioeconomic status is in itself a risk factor for preterm birth. Lack of coverage or difficulty in obtaining access to transvaginal ultrasound should not prevent obstetricians from requesting this procedure when it is indicated.

There are many Brazilian maternity hospitals that include universal ultrasound screening of the cervical length to try to predict preterm birth, such as the Maternity school of Rio de Janeiro University, and Helda Gerdau Johannpetter Maternity, at Moinhos de Vento Hospital, in Porto Alegre/RS. Although gynecology and obstetrics societies do not have specific protocols addressing prematurity screening, and Febrasgo (Brazilian Federation of Gynecology and Obstetrics)^(19)^ expressed support for this measure to predict the risk of preterm birth.

Indeed, one might argue that it should be incorporated into routine prenatal care of pregnant women with lower socioeconomic status. The stability in preterm birth rates despite great advances in perinatal medicine may be due to the fact that we are not universally screening a population considered to be at high risk.

The limitations of our study are those inherent to research involving self-report questionnaires, voluntary response bias, and convenience sampling bias, which can over- or underestimate actual screening and treatment practices.

## Conclusion

Although the relationship between short cervical length diagnosed up to gestational age 24 weeks and preterm birth is well established, there are still many controversies surrounding which patients should be screened, when such screening should take place, and what is the most appropriate screening modality. The obstetricians who completed our survey reported practices consistent with the literature regarding screening for preterm birth, but there is still a need for greater uniformity in practice if we are to assess the true impact of screening on preterm birth and its consequences in our population, particularly regarding indications for and use of progesterone in the prevention of preterm birth. Developing a protocol to guide management of pregnant women in Brazil in this respect could be an effective means of reducing the alarming incidence of preterm birth and its myriad public health implications. Further research is needed to ascertain true incidence and prevalence and to elucidate the reasons for the high rate of spontaneous preterm birth observed in the country.

## References

[1] World Health Organization (WHO) (2012). Born too soon: the global action report on preterm birth.

[2] To MS, Skentou CA, Royston P, Yu CK, Nicolaides KH (2006). Prediction of patient-specific risk of early preterm delivery using maternal history and sonographic measurement of cervical length: a population-based prospective study. Prediction of patient-specific risk of early preterm delivery using maternal history and sonographic measurement of cervical length: a population-based prospective study.

[3] Owen J, Yost N, Berghella V, Thom E, Swain M, Dildy GA (2001). Mid-trimester endovaginal sonography in women at high risk for spontaneous preterm birth. JAMA.

[4] Romero R, Conde-Agudelo A, Da Fonseca E, O’Brien JM, Cetingoz E, Creasy GW (2018). Vaginal progesterone for preventing preterm birth and adverse perinatal outcomes in singleton gestations with a short cervix: a meta-analysis of individual patient data. Am J Obstet Gynecol.

[5] Instituto Brasileiro de Geografia e Estatística (IBGE) (2021). Rio Grande do Sul: População.

[6] Governo do Estado do Rio Grande do Sul (2022). RS registra menor taxa de crescimento vegetativo da série histórica em 2021.

[7] Martell B, DiBenedetti DB, Weiss H, Zhou X, Reynolds M, Berghella V (2018). Screening and treatment for short cervical length in pregnancy: a physician survey in the United States. Arch Gynecol Obstet.

[8] Einerson BD, Grobman WA, Miller ES (2016). Cost-effectiveness of risk-based screening for cervical length to prevent preterm birth. Am J Obstet Gynecol.

[9] Werner EF, Hamel MS, Orzechowski K, Berghella V, Thung SF (2015). Cost-effectiveness of transvaginal ultrasound cervical length screening in singletons without a prior preterm birth: an update. Am J Obstet Gynecol.

[10] Berghella V, Palacio M, Ness A, Alfirevic Z, Nicolaides KH, Saccone G (2017). Cervical length screening for prevention of preterm birth in singleton pregnancy with threatened preterm labor: systematic review and meta-analysis of randomized controlled trials using individual patient-level data. Cervical length screening for prevention of preterm birth in singleton pregnancy with threatened preterm labor: systematic review and meta-analysis of randomized controlled trials using individual patient-level data.

[11] Navathe R, Saccone G, Villani M, Knapp J, Cruz Y, Boelig R (2019). Decrease in the incidence of threatened preterm labor after implementation of transvaginal ultrasound cervical length universal screening. J Matern Fetal Neonatal Med.

[12] Romero R, Yeo L, Chaemsaithong P, Chaiworapongsa T, Hassan SS (2014). Progesterone to prevent spontaneous preterm birth. Progesterone to prevent spontaneous preterm birth.

[13] Norman JE (2020). Progesterone and preterm birth. Progesterone and preterm birth.

[14] Norman JE, Marlow N, Messow CM, Shennan A, Bennett PR, Thornton S (2016). Vaginal progesterone prophylaxis for preterm birth (the OPPTIMUM study): a multicentre, randomised, double-blind trial. Lancet.

[15] Jarde A, Lutsiv O, Park CK, Beyene J, Dodd JM, Barrett J (2017). Effectiveness of progesterone, cerclage and pessary for preventing preterm birth in singleton pregnancies: a systematic review and network meta-analysis. BJOG.

[16] Pagani G, Stagnati V, Fichera A, Prefuno F (2016). Cervical length at mid-gestation in screening for preterm birth in twin pregnancy. Cervical length at mid-gestation in screening for preterm birth in twin pregnancy.

[17] Romero R, Conde-Agudelo A, El-Refaie W, Rode L, Brizot ML, Cetingoz E (2017). Vaginal progesterone decreases preterm birth and neonatal morbidity and mortality in women with a twin gestation and a short cervix: an updated meta-analysis of individual patient data. Ultrasound Obstet Gynecol.

[18] Rehal A, Benkő Z, De Paco Matallana C, Syngelaki A, Janga D, Cicero S (2021). Early vaginal progesterone versus placebo in twin pregnancies for the prevention of spontaneous preterm birth: a randomized, double-blind trial. Am J Obstet Gynecol.

[19] Federação Brasileira das Associações de Ginecologia e Obstetrícia (FEBRASGO) (2021). Ultrassonografia morfológica do segundo trimestre.

